# Spontaneous pneumomediastinum incidence and clinical features in non-intubated patients with COVID-19

**DOI:** 10.6061/clinics/2021/e2959

**Published:** 2021-09-06

**Authors:** Miktat Arif Haberal, Erkan Akar, Ozlem Sengoren Dikis, Mehmet Oguzhan Ay, Hakan Demirci

**Affiliations:** I Department of Thoracic Surgery, Bursa Yuksek Ihtisas Training and Research Hospital, Health Sciences University, Bursa, Turkey.; II Department of Pulmonary Medicine, Bursa Yuksek Ihtisas Training and Research Hospital, Health Sciences University, Bursa, Turkey.; III Department of Emergency Medicine, Bursa Yuksek Ihtisas Training and Research Hospital, Health Sciences University, Bursa, Turkey.; IV Department of Family Medicine, Bursa Yuksek Ihtisas Training and Research Hospital, Health Sciences University, Bursa, Turkey.

**Keywords:** Penumomediastinum, COVID-19, Thoracic Surgery

## Abstract

**OBJECTIVES:**

To evaluate the presentation characteristics and disease course of seven patients with COVID-19 who spontaneously developed pneumomediastinum without a history of mechanical ventilation.

**METHODS:**

A total of seven non-intubated patients with COVID-19, of age ranging from 18-67 years, who developed spontaneous pneumomediastinum between 01 April and 01 October 2020 were included in the study. Patients' demographic data, clinical variables, and laboratory values were examined. Spontaneous pneumomediastinum was evaluated using posteroanterior chest radiography and thorax computed tomography.

**RESULTS:**

During the research period, 38,492 patients reported to the emergency department of our hospital with COVID-19 symptoms. Of these, spontaneous pneumomediastinum was detected in seven patients who had no previous history of intubation. Chronic obstructive pulmonary disease (2/7) and asthma bronchiale (2/7) were determined as the most common causes of comorbidity.

**CONCLUSIONS:**

In our study, the frequency of spontaneous pneumomediastinum developing without pneumothorax was found to be high in non-intubated patients. Whether this is related to the nature of the disease or it is a result of the increase in cases diagnosed incidentally owing to the increasing use of low-dose computed tomography should be explored in further studies.

## INTRODUCTION

Pneumomediastinum (PM) is the collection of free air within the mediastinum. It was first described by Hamman in 1939. PM was defined as the entry of free air from the peribronchovascular tissue to the mediastinum (Macklin effect) as a result of tears in the terminal alveoli of the lung due to increased intraalveolar pressure (1,2).

Although spontaneous pneumomediastinum (SPM) is rare, it mostly occurs as a result of the rupture of pulmonary alveoli in healthy young men. Predisposing factors for spontaneous alveolar rupture include asthma, chronic obstructive lung disease (COPD), interstitial lung diseases, tobacco use, continuous use of legal or illegal inhaler drugs, vomiting, cough, upper respiratory tract infections, constipation, physical exercise, respiratory distress syndrome and rarely, events or conditions such as balloon blowing, playing wind instruments, or convulsions (3-5).

The most common symptoms encountered in the clinic are dyspnea, chest pain, neck pain, and subcutaneous emphysema. Hearing the crackling sound caused by heart crest beats during auscultation on the front of the chest is known as the Hamman sign (6-8). While SPM is observed as a complication of mechanical ventilation in patients with viral pneumonia, the development of this condition in non-intubated patients suggests an alternative etiology (9).

Our study aims to evaluate the presentation characteristics and course of the disease in seven patients with SPM who were diagnosed with COVID-19 without endotracheal intubation in the hospital or before and did not develop pneumothorax during the course of treatment.

## METHODS

### Ethical Statement

The authors are accountable for all aspects of the work in ensuring that questions related to the accuracy or integrity of any part of the work are appropriately investigated and resolved. The study conforms to the provisions of the Declaration of Helsinki (as revised in 2013). This retrospective study was approved by the Institutional Review Board (2011-KAEK-25 2020/10-13) and the informed consents were waived by approval of Institutional Review Board.

The files of seven patients who were confirmed to be COVID-19 positive by reverse transcriptase-polymerase chain reaction (RT-PCR) test, without a history of mechanical ventilation, and were diagnosed with SPM by consultation to thoracic surgery and chest diseases specialists between April 01 2020 and October 01 2020 were reviewed. The demographic characteristics and clinical and laboratory data of the patients included in the study were noted separately for each.

Demographic parameters included age, gender, smoking status, and comorbidity. Patients' body temperature, arterial oxygen saturation (% SpO_2_), and mean arterial blood pressure (mmHg) at the time of first admission were measured. Symptoms such as cough and shortness of breath were recorded at admission. Symptom onset dates were noted. Hospitalization parameters including invasive ventilation and mortality details were collected.

Finally, the laboratory values collected include white blood cell (WBC) count, absolute lymphocyte count, lactate dehydrogenase (LDH), hypersensitive C-reactive protein (CRP), D-dimer, ferritin, fibrinogen, aspartate aminotransferase (AST), and alanine aminotransferase (ALT) values. Posteroanterior (PA) chest radiographs and low-dose thorax computed tomography (CT) images of all patients were examined. The clinical, laboratory, and demographic characteristics of the patients at the first admission were tabulated and presented (Table 1 and 2).

## RESULTS

During the research period, 38,492 patients reported to the emergency department of our hospital with COVID-19 symptoms. Seven patients with a diagnosis of SPM and positive RT-PCR test results were included in the study. The patients included five males and two females, with the age distribution of 18-67 years and mean age of 41.5 years (Table 1). None of our patients received invasive mechanical ventilation treatment before diagnosis. Three of our patients had a history of smoking. In terms of comorbidity, the most common were COPD (2/7), asthma bronchiale (2/7), and hypertension (1/7). There were no comorbid diseases in two cases. The mean body temperature of the patients at their first admission to the emergency department was 37.0°C (36.5-37.7°C), and the mean oxygen saturation was 93.5% (95-100%). The blood pressure of the patients was between 120/70-160/90 mmHg. The most common symptoms, in order of frequency, were cough, fever, and dyspnea.

**Figure 1:**
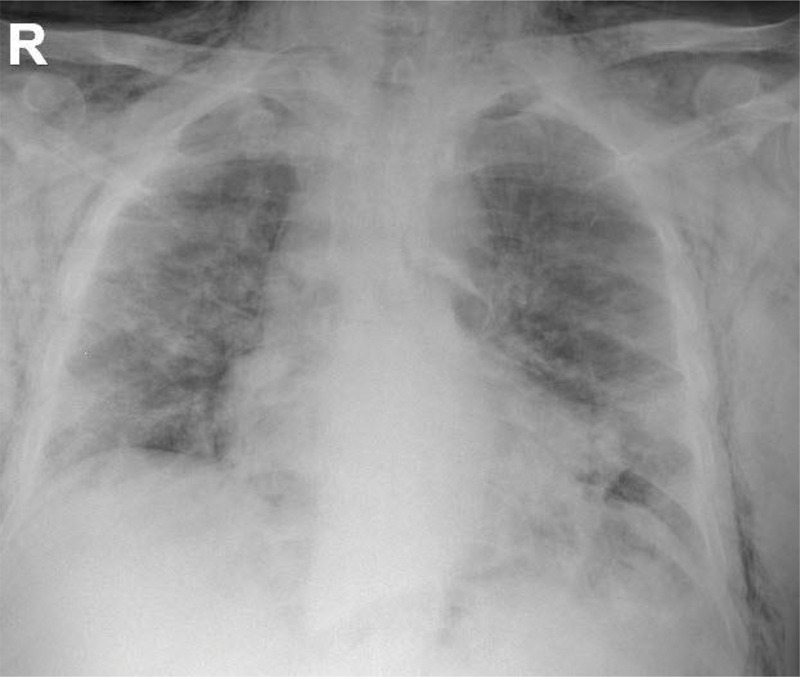
Diffuse subcutaneous emphysema and bilateral parenchymal diffuse ground glass appearance on posteroanterior chest radiography.

When the initial laboratory data of the cases were examined, fibrinogen was detected in the range of 360-574 mg/dL, WBC 8.29-15.4 mcl, lymphocyte % count 16.5-30.2%, LDH 290-610 U/L, CRP 9.4-75 mg/L, ferritin 118-610 ng/mL, AST 20.9-47.1 U/L, ALT 11-51.2 U/L, and D-Dimer 0.29-4.1 μg/mL (Table 2). When imaging methods were examined, SPM was diagnosed in the PA chest radiography and thorax CT (Figure 1) of one of our patients at the first admission to the hospital, while the diagnosis was made in our other patients after hospitalization. Subcutaneous emphysema was observed in all our patients with a diagnosis of SPM. The patients received oxygen therapy at 5-10/L/min. According to laboratory tests, thorax CT, and clinical evaluation results, COVID-19 viral infection treatment was arranged in accordance with the recommendations of pulmonologists.

Patients with increased inflammation parameters such as CRP >5 mg/L and D-dimer levels (>0.5 μg/mL) were administered low molecular weight heparin (LMWH) at a dose of 0.5 mg/kg (twice a day) and Favipiravir 200 mg at 2×1600 mg loading and 2×600 mg maintenance doses. Patients whose general condition was stable and complaints and inflammatory parameters were decreased were discharged. Patients with D-dimer values above 0.5 μg/mL at discharge were administered a single dose of LMWH (40 mg, once a day) for 30 days. Patients with pulmonary involvement during their hospitalization were given moxifloxacin (400 mg once a day) or amoxicillin (1,000 mg twice daily) for 1 week during the discharge period. After discharge, these patients were monitored at home for 14 days by radiation teams (the team monitoring COVID-19 patients at home). One of our patients died on the 20^th^ day of treatment due to multiple organ failure.

## DISCUSSION

Considering that SPM is extremely rare in the general population (1:7000 to 1:45000) (10), we observed this ratio to be 1:5.498 (7:38492) in patients who applied to our hospital due to COVID-19. This rate is the highest we have encountered in the literature. This high rate may be related to the course of the COVID-19 disease; however, another possibility is that the more frequent use of low-dose CT examinations resulted in incidental diagnoses.

Common symptoms seen in COVID-19 patients are fever, cough, severe headache, sore throat, myalgia, fatigue, diarrhea, and vomiting (11,12). In our study, the presenting symptoms of the patients were cough, fever, and dyspnea. In another study, severe hypoxia was observed in 6/11 of the patients who presented with SPM at the time of admission (13). In our study, the oxygen saturation of the patients was not low except in one case.

SPM is generally observed in the young male population (1). In cases diagnosed with COVID-19, SPM was observed with a higher rate among males. This may be related to the fact that COVID-19 disease is more common in the male population. The body temperature of the patients at the time of admission was subfebrile. However, high fever cases were reported in a sample study (13).

As COVID-19 can be transmitted through respiratory droplets and contact, transmission and spread rates are high. Infection by other viruses such as influenza A or B can cause symptoms similar to COVID-19. It is difficult to distinguish COVID-19 from other infections, especially during the flu season (14). SPM is rare in viral pneumonia. It has been previously reported in cases with COVID-19 pneumonia associated with severe acute respiratory failure syndrome (15,16). Although the exact mechanism is unknown, it is thought that in severe COVID-19 pneumonia, increased alveolar pressure and widespread alveolar damage increase the susceptibility of the alveoli to rupture. The prevalence of cough in these patients may play a role as a predisposing factor in the occurrence of SPM (17). To date, there is little literature on the occurrence of SPM in patients with COVID-19 who have not received mechanical ventilation therapy.

**Figure 2:**
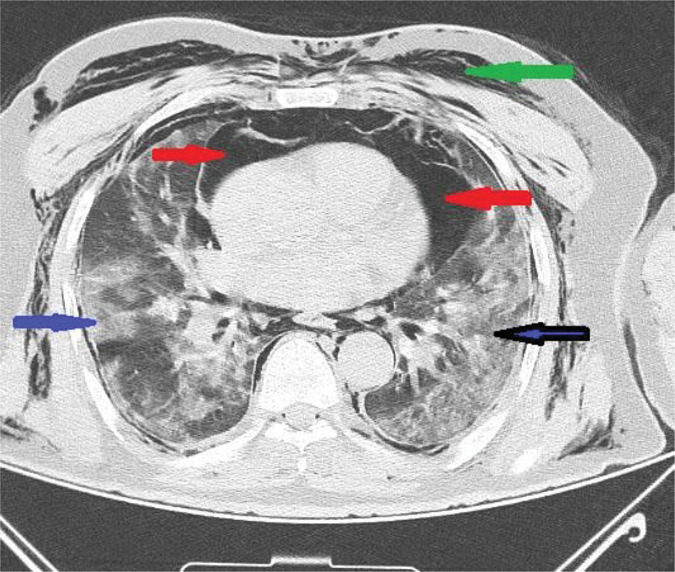
Thorax computed tomography shows diffuse air density around the heart (red arrow), ground glass appearance in bilateral parenchyma (blue arrow) and diffuse air entrapment under the skin (green arrow).

In COVID-19 infection, the development of SPM is considered a possible indicator of the deterioration of the patient's general condition. Owing to its high sensitivity and ease of use, low-dose thorax CT is an essential screening tool for patients with suspected COVID-19. The most common CT finding in COVID-19 pneumonia is ground glass appearance in the subpleural regions of the lower lobes (18). During the research period, we obtained low-dose CT images from every patient with COVID-19. In 4/7 (57.1%) of our cases, CT findings indicated that the lesions were localized in the lower lobes (Figure 2).

COVID-19 has caused a dramatic increase in the number of patients requiring intensive care. While mediastinal emphysema and pneumothorax are well-known complications of mechanical ventilation, it has been emphasized that mediastinal emphysema may be associated with COVID-19 pneumonia in patients without a history of mechanical ventilation (10). Before the diagnosis of SPM, our patients only received oxygen therapy with a high-flow nasal cannula, and none of them had a history of mechanical ventilation.

SPM is a rare condition most commonly caused by medical conditions such as asthma, COPD, infections, and mechanical ventilation. Despite its low incidence, it should be considered in the differential diagnosis of acute chest pain (19). It was determined that two of our cases were treated for asthma bronchiale and two for COPD. Extensive subcutaneous emphysema including the neck region and anterior thorax wall was detected in one of our patients upon arrival in the emergency department, and in the evaluation of vital signs by pulse oximetry, SpO_2_ was found to be 88%. Considering the patient’s poor general condition, the patient was intubated and taken to the intensive care unit. Fiberoptic bronchoscopy and esophagoscopy were performed to investigate the SPM etiology of the patient, and the pathology of the digestive and tracheobronchial systems were excluded. SPM should be monitored carefully as it may cause respiratory pathology.

In conclusion, our study examining cases of COVID-19 infection demonstrated that the frequency of SPM development without pneumothorax was found to be high in non-intubated patients. Further studies may reveal whether this is related to the nature of the disease or the increasing diagnosis due to the excessive use of low-dose CT. In addition, it should be considered by the clinicians that SPM may develop rarely in patients with viral pneumonia due to COVID-19.

## AUTHOR CONTRIBUTIONS

All authors contributed to the manuscript. Haberal MA, Akar E and Dikis OS were responsible for the study conception and design. Ay MO and Demirci H were responsible for the administrative support. Haberal MA, Akar E and Ay MO were responsible for the provision of study materials. Haberal MA, Akar E and Dikis OS were responsible for the collection and assembly of data. Haberal MA, Ay MO and Demirci H were responsible for the data analysis and interpretation. All of the authors were responsible for the manuscript writing and approved its final version.

## Figures and Tables

**Table 1:** Demographic characteristics of patients.

Patient Number	1	2	3	4	5	6	7
Age (year)	67	58	49	18	38	18	43
Gender	M	M	M	F	M	M	F
Comorbidity	COPD	COPD	Hypertension	Asthma	-	-	Asthma
Symptoms	Cough Dyspnea Fever	Cough Dyspnea Joint pain	Cough Fever Headache	Cough Dyspnea Joint pain	Cough Dyspnea Fever	Cough Dyspnea Fever	Cough Dyspnea Fever
Body temperature (°C)	37.0	36.8	37.4	36.6	37.7	37.0	37.6
Saturation (%)	95	94	95	88	94	93	96
Blood pressure (mmHg)	140/80	130/70	160/90	120/80	130/70	110/80	120/70
Smoking history	Yes	Yes	Yes	No	Yes	No	No
Ground glass opacities detected on the thorax CT	Yes	Yes	No	Yes	No	No	Yes
Spontaneous pneumomediastinum occurrence time (day)	1	3	5	10	8	7	4
Treatment time (day)	1	20	15	10	10	12	10

M: Male; F: Female; COPD: chronic obstructive pulmonary disease.

**Table 2:** Laboratory data of patients.

Patient Number	1	2	3	4	5	6	7
WBC (3.5-10.5 mcl)	8.29	11.5	10.7	9.8	15.4	13.5	12.7
Lymphocyte % (19.3-42.5)	25.7	19.4	20.3	30.2	16.5	17.9	18.4
CRP (0-0.5 mg/L)	10.4	11.2	9.4	75.0	65.4	10.3	35.2
D-Dimer (0-0.5 μg/mL)	0.29	1.2	1.1	3.2	4.1	0.9	1.9
LDH (135-225 U/L)	290	310	380	610	520	279	410
AST (0-40 U/L)	20.9	35.0	47.1	42.2	39.1	22.0	35.2
ALT(5-41 U/L)	45.3	38.4	51.2	38.1	40.4	11.0	37.4
Ferritin (30-400 ng/mL)	533	450	480	400	610	118	380
Fibrinogen (180-350 mg/dL)	574	410	430	360	480	388	490

WBC: White blood cell; CRP: C-Reactive Protein; LDH: Lactate dehydrogenase; AST: Aspartate aminotransferase; ALT: Alanine aminotransferase.

## References

[1] Macia I, Moya J, Ramos R, Morera R, Escobar I, Saumemch J (2007). Spontaneous pneumomediastinum: 41 cases. Eur J Cardiothorac Surg.

[2] Kouritas VK, Papagiannopoulos K, Lazaridis G, Bakas S, Mpoukovinas I, Karavasilis V (2015). Pneumomediastinum. J Thorac Dis.

[3] Marasco RD, Loizzi D, Ardò NP, Fatone FN, Sollitto F (2018). Spontaneous Pneumomediastinum After Electronic Cigarette Use. Ann Thorac Surg.

[4] Fishman A, Elias J, Fishman J, Grippi M, Senior R, Pack A (2008). Fishman's Pulmonary Disease and Disorders. 4.

[5] Freixinet J, García F, Rodríguez PM, Santana NB, Quintero CO, Hussein M (2005). Spontaneous pneumomediastinum long-term follow-up. Respir Med.

[6] Ryoo JY (2012). Clinical analysis of spontaneous pneumomediastinum. Tuberc Respir Dis (Seoul).

[7] Jougon JB, Ballester M, Delcambre F, Mac Bride T, Dromer CE, Velly JF (2003). Assessment of spontaneous pneumomediastinum: experience with 12 patients. Ann Thorac Surg.

[8] Kira K, Inokuchi R, Maehare H, Tagami S (2016). Spontaneous pneumomediastinum. BMJ Case Rep.

[9] Manna S, Wruble J, Maron SZ, Toussie D, Voutsinas N, Finkelstein M (2020). COVID-19: A Multimodality Review of Radiologic Techniques, Clinical Utility, and Imaging Features. Radiol Cardiothorac Imaging.

[10] Meireles J, Neves S, Castro A, França M (2011). Spontaneous pneumomediastinum revisited. Respir Med CME.

[11] Sun R, Liu H, Wang X (2020). Mediastinal Emphysema, Giant Bulla, and Pneumothorax Developed during the Course of COVİD-19 Pneumonia. Korean J Radiol.

[12] Chen N, Zhou M, Dong X, Qu J, Gong F, Han Y (2020). Epidemiological and clinical characteristics of 99 cases of 2019 novel coronavirus pneumonia in Wuhan, China: a descriptive study. Lancet.

[13] Manna S, Maron SZ, Cedillo MA, Voutsinas N, Toussie D, Finkelstein M (2020). Spontaneous subcutaneous emphysema and pneumomediastinum in non-intubated patients with COVID-19. Clin Imaging.

[14] Ding Q, Lu P, Fan Y, Xia Y, Liu M (2020). The clinical characteristics of pneumonia patients coinfected with 2019 novel coronavirus and influenza virus in Wuhan, China. J Med Virol.

[15] Zhao W, Zhong Z, Xie X, Yu Q, Liu J (2020). Relation Between Chest CT Findings and Clinical Conditions of Coronavirus Disease (COVID-19) Pneumonia: A Multicenter Study. AJR Am J Roentgenol.

[16] Chu CM, Leung YY, Hui JY, Hung IF, Chan VL, Leung WS (2004). Spontaneous pneumomediastinum in patients with severe acute respiratory syndrome. Eur Respir J.

[17] Singh A, Bass J, Lindner DH (2020). Rare Complication of Pneumomediastinum and Pneumopericardium in a Patient with COVID-19 Pneumonia. Case Rep Pulmonol.

[18] Bernheim A, Mei X, Huang M, Yang Y, Fayad ZA, Zhang N (2020). Chest CT Findings in Coronavirus Disease-19 (COVID-19): Relationship to Duration of Infection. Radiology.

[19] Çakmak M, Yüksel M, Kandemir MN (2016). Analysis of Patients with Spontaneous Pneumomediastinum. Turk Thorac J.

